# Supplementary material for: Chikungunya Virus E2 Structural Protein B-Cell Epitopes Analysis
Source: Viruses. 2022 Aug 22;14(8):1839. doi: 10.3390/v14081839 (PMC9414204; doi:10.3390/v14081839)
Supplement: Supplementary file 1 [file viruses-14-01839-s001.zip › viruses-1860318-supplementary.pdf]

Supplementary information for:

Chikungunya Virus E2 structural protein B-cell epitopes analisys

João Paulo da Cruz Silva, Marielton dos Passos Cunha, Shahab Zaki Pour, Vitor Renaux Hering, Daniel Ferreira de Lima Neto, Paolo Marinho de Andrade Zanotto[#]

# Corresponding author:
pzanotto@usp.br (PMAZ)

Contents:

- Supplementary Figure S1
- Supplementary Figure S2
- Supplementary Figure S3

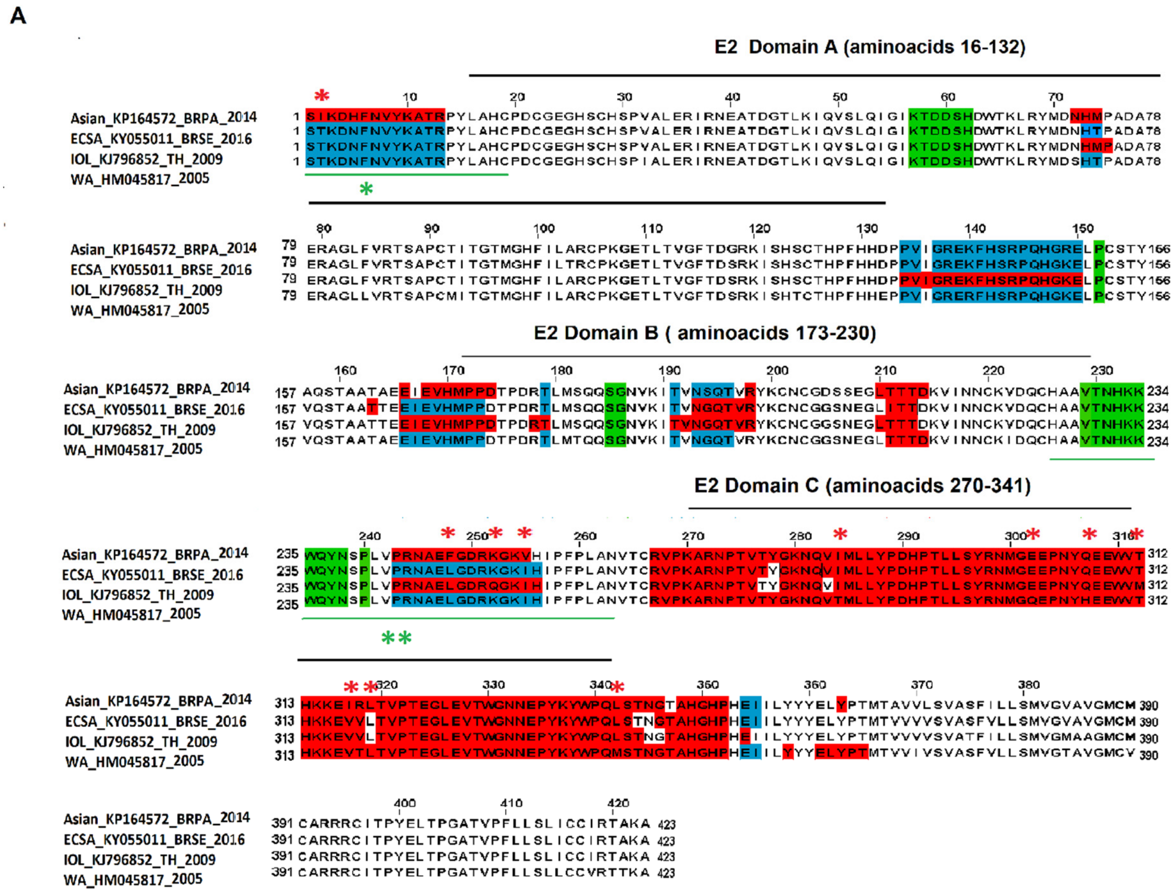

Supplementary Figure S1. Conformational epitopes prediction for Chikungunya virus E2 Protein. Multiple alignment of E2 protein sequences corresponding to the four genotypes with highlighted epitopes conformational prediction. On green epitopes are conserved among all genotypes. Blue epitopes are common to more than one genotype. On red single epitopes of each genotype; *- Amino acid changes in the regions predicted as epitopes. *, ** CHIKV epitopes already identified and described.

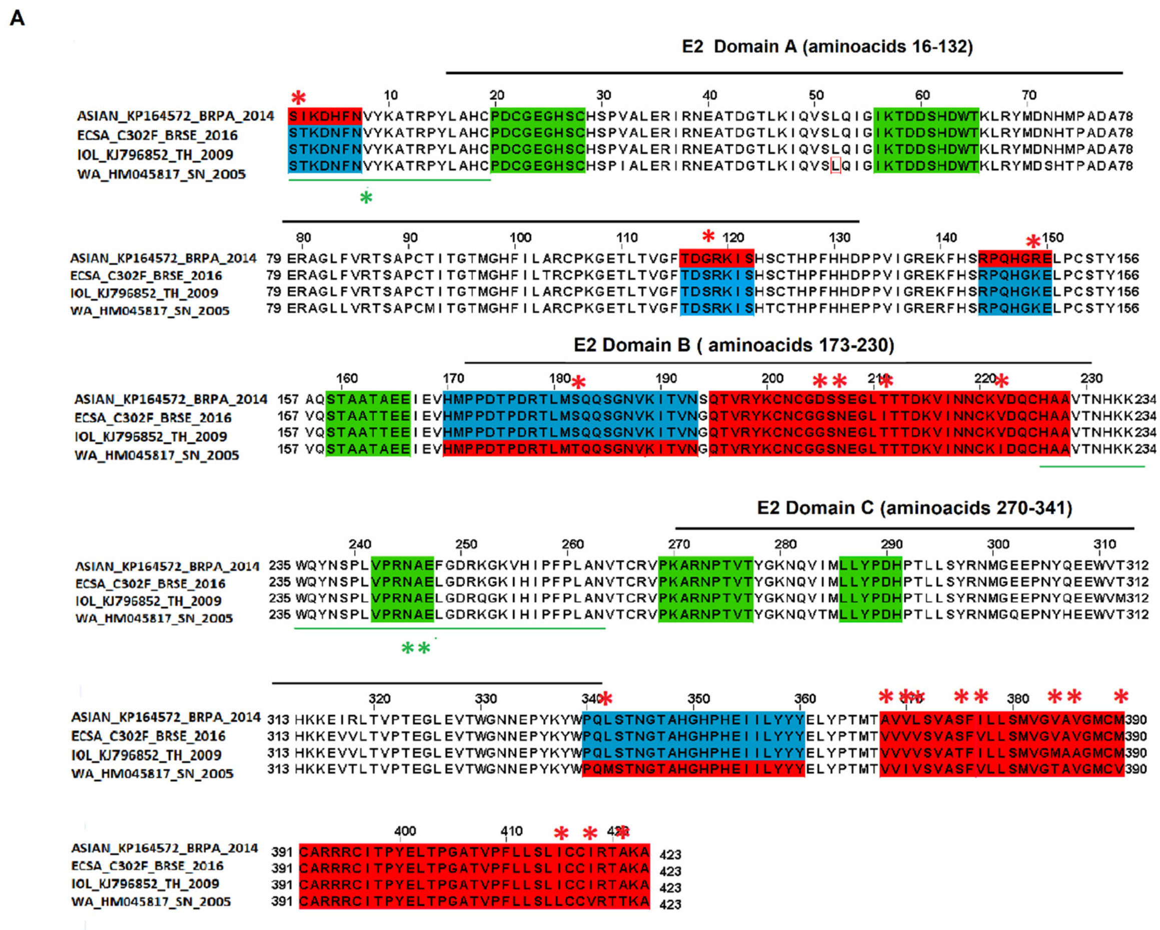

Supplementary Figure S2. Linear epitopes prediction for Chikungunya virus E2 Protein. Multiple alignment of E2 protein sequences corresponding to the four genotypes with highlighted epitopes linear prediction. On green epitopes are conserved among all genotypes. Blue epitopes are common to more than one genotype. On red single epitopes of each genotype; *- Amino acid changes in the regions predicted as epitopes. *, ** CHIKV epitopes already identified and described.

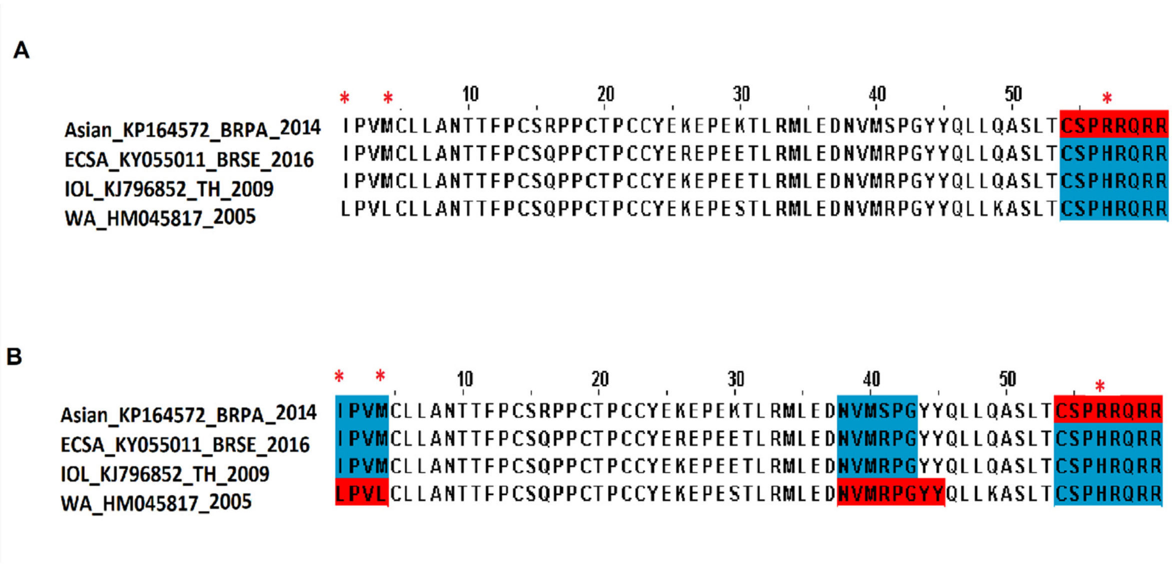

Supplementary Figure S3. Conformational and Linear epitopes prediction for Chikungunya virus E3 Protein. Multiple alignment of E3 protein sequences corresponding to the four genotypes with highlighted conformational (**A**) and linear (**B**) epitopes predictions. Blue epitopes are common to more than one genotype. On red single epitopes of each genotype; *- Amino acid changes in the regions predicted as epitopes.
